# Relevant vs non-relevant subspecialist for patients hospitalised in internal medicine at a local hospital: which is better? A retrospective cohort study

**DOI:** 10.1186/s12913-022-08761-1

**Published:** 2022-11-14

**Authors:** Øyvind Berg, Ulf Hurtig, Aslak Steinsbekk

**Affiliations:** 1 Faculty of Medicine and Health Sciences, Department of Public Health and Nursing, Norwegian University of Science and Technology, 7491 Trondheim, Norway; 2 Innlandet Hospital Trust, Division Tynset, 2500 Tynset, Norway

**Keywords:** Subspecialisation, Internal medicine, Treatment outcome, Readmissions, In-hospital mortality, Length of stay

## Abstract

**Background:**

Studies of the treatment of patients in-hospital with a specific diagnosis show that physicians with a subspecialisation relevant to this diagnosis can provide a better quality of care. However, studies including patients with a range of diagnoses show a more negligible effect of being attended by a relevant subspecialist. This project aimed to study a more extensive set of patients and diagnoses in an environment where the subspecialist present could be controlled. Thus, this study investigated whether being attended by a physician with a subspeciality relevant to the patient’s primary diagnosis was prospectively associated with readmission, in-hospital mortality, or length of stay compared to a physician with a subspeciality not relevant to the patient’s primary diagnosis.

**Methods:**

We have conducted a retrospective register-based study of 11,059 hospital admissions across 9 years at a local hospital in south-eastern Norway, where it was possible to identify the physician attending the patients at the beginning of the stay. The outcomes studied were emergency readmissions to the same ward within 30 days, any in-hospital mortality and the total length of stay. The patients admitted were matched with the consultant(s) responsible for their treatment. Then, the admissions were divided into two groups according to their primary diagnosis. Was their diagnosis within the subspeciality of the attending consultant (relevant subspecialist) or not (non-relevant subspecialist). The two groups were then compared using bivariable and multivariable models adjusted for patient characteristics, comorbidities, diagnostic group and physician sex.

**Results:**

A relevant subspecialist was present during the first 3 days in 8058 (73%) of the 11,059 patient cases. Patients attended to by a relevant subspecialist had an odds ratio (OR) of 0.91 (95% confidence interval 0.76 to 1.09) for being readmitted and 0.71 (0.48 to 1.04) for dying in the hospital and had a length of stay that was 0.18 (− 0.07 to 0.42) days longer than for those attended to by a non-relevant subspecialist.

**Conclusions:**

This study found that patients attended by a relevant subspecialist did not have a significantly different outcome to those attended by a non-relevant subspecialist.

**Supplementary Information:**

The online version contains supplementary material available at 10.1186/s12913-022-08761-1.

## Background

Being attended by a physician with a speciality covering the patient’s current diagnosis has been shown to give favourable outcomes for specific diagnoses [1, 2]. A proposed reason is that the more specialised physicians use a more specific approach when treating diagnoses within their field of speciality [3]. Conversely, the subspecialists might be less efficient when practising outside their speciality areas and provide a lower quality of care [3, 4]. Thus, the question arises: How does it affect patients when the physicians cover a broader field than their subspeciality, e.g. on-call at nights, weekends or on general wards?

A lower specialist intensity was hypothesised to be the reason for the worse patient outcome found among patients admitted at weekends [5–7]. However, a more recent study found no correlation between specialist intensity and mortality [8]. Another study from the USA even found lower mortality for acute cardiac conditions during national cardiology meetings when the specialist intensity presumably was lower [9].

Physician experience and patient outcomes have been studied by, amongst others, McAlister et al., without finding a relation between them [10]. Bai et al. studied patient outcomes when the attending physician at discharge/death was a general internist compared to a specialist and found that the generalists’ patients had shorter hospitalisations but the same readmission and mortality rates [11]. Weingarten et al. studied four subspecialities treating four conditions, each relevant for their subspeciality [4]. They found that when the “physician of record” was practising within their field of subspeciality, there was a shorter length of stay and lower mortality than when practising outside their field of subspeciality. Most recently, Smyth et al. studied the effects of an admission program where a relevant subspecialist’s team attended the patient instead of the team of the admitting subspecialist. They found a prolonged length of stay but without significant impact on mortality and readmissions when patients were assigned to a relevant subspecialist [12].

There is still a need for more studies in this area, especially with a design that more consistently matches the diagnosis of the patient and the subspeciality of the attending physician who is likely to have the most influence over the outcome. The earlier studies have taken place in larger hospitals where other consultants could be interfering without being the discharging physician/physician of record. This study utilises data from a hospital with a staffing model with only two consultants present per week to study the impact of attending consultants’ subspeciality more precisely on the admitted patients’ outcomes.

The aim of this study was therefore to investigate if being attended at the start of the stay by a physician with a subspeciality relevant to the patient’s primary diagnosis—compared to a physician with a subspeciality not relevant to the patient’s primary diagnosis—was prospectively associated with readmission rate, length of stay, and in-hospital mortality.

## Method

### Design

This study was a retrospective cohort study using registry data about patient admissions and rosters of physicians from a small-sized local hospital for the period 2005–2017. All methods were conducted following relevant guidelines and regulations.

### Setting

The study hospital is situated in the interior part of south-eastern Norway, covering a population of around 25,000 persons [13]. It has emergency functions in orthopaedics, general surgery, and internal medicine. In addition, elective treatment and outpatient services are offered in urology and plastic surgery.

The internal medicine department had 20 ordinary beds (from November 2017, 16 beds), 4 beds in a high dependency unit, and outpatient service. It is staffed with one consultant on weekends and holidays and two on weekdays. The yearly inpatient admission has been around 1700 patients.

As a solution to the difficulty of attracting specialists to live and work in rural Norway, the hospital has for more than 20 years employed consultants who work for “1–2 weeks with clinical activity and continuous duty at the hospital, and 2–4 weeks of independent working time for administrative duties, professional updating and holiday/spare time” [14].

The size of the consultant staff has been stable over time. All consultants are employed with time on and off, as described above. In 2017, the consultants filled 6.2 full-time equivalent positions in medicine, 8.5 in general surgery and orthopaedics, 3 in anaesthesia and 3 in radiology. In medicine, 5 consultants worked more than 9 weeks each (more than 0.5 full-time equivalent); these 5 covered 71% of the shifts. The remaining 29% were covered by 11 consultants working 5 weeks or less. In addition, there is one position as a speciality registrar in internal medicine and eight as foundation doctors (rotating between medicine and surgery).

The consultants work either a 5-day shift (Monday–Friday with two 24 h shifts on-call) or a 7-day shift (Friday–Friday being on-call Friday–Monday in addition to two 24 h shifts). At weekends, the consultant present attends the ward and is on call. On weekdays, the two consultants divide the ward between them and work in the outpatient clinic, with one of them on call. Only two consultants are present each week.

After admission to the ward from the emergency room, the patient will be assigned to one of the two consultants on weekdays or the one present on weekends, usually to the one most competent in the primary diagnosis/complaint. Patients admitted for the last 24 h are presented to the consultants and interns present each morning. Patients admitted Friday–Sunday who are still admitted are presented on Monday.

### Participants

Two types of participants, patients and physicians, were included. The criteria for inclusion in the study were patients with emergency admission to the internal medicine ward between 2005 and 2017 where the physician(s) present could be identified. Patients were excluded if they lacked a diagnostic code within internal medicine at discharge.

Physicians were included if they could be matched to a patient admitted to the internal medicine ward either by being on call at admission or by attending the ward at least one of the first 3 days of stay.

### Sample size calculation

The hospital as a whole has had a readmission rate of 15–17% [15]. A total of 10,004 patients had to be enrolled to have a power of 80% and alpha of 0.05 of detecting a difference of ±1% in readmission between the two groups [16]. The yearly admission rate in internal medicine has been around 1700, and it was decided to include data for 13 years (from 2005 to 2017) as missing data was expected for some years.

### Data collection and variables

Staff at the hospital provided de-identified data for patients admitted to the internal medicine ward and the consultants’ rosters for the study period.

The patient data included a de-identified ID, patient’s age, gender, state at discharge (dead or alive), urgency (elective or emergency), primary and secondary diagnoses at discharge, and the time of admission and discharge. Setting diagnoses, including deciding on the primary diagnosis, is done by the discharging physician.

The physician data included when they had been present according to rosters updated at the end of each year to show their actual presence, the subspeciality, and sex, all corroborated by the hospital’s chief of medicine, who was working at the hospital during the entire study period.

The rosters for 2010 and 2011 were not found, and neither was the updated roster for 2009. Patient data were therefore collected for 2005–2008 and 2012–2017. The available variables differed somewhat. The required data were present for all years, except 2005, 2006, and 2007. Age, sex, time of admission and discharge, and state at discharge was lacking for 2005. For 2006 and 2007, the state at discharge was missing. For 2007, information about urgency was also missing. The available data were used where relevant for specific analysis.

### Matching patients with attending consultants

A patient was coded as attended by a relevant subspecialist if there was a match between the patient’s primary diagnosis at discharge and the subspeciality of either the consultant on call at admission or a consultant tending the ward during the first 3 days after admission.

Matching diagnosis and subspeciality was complicated. To our knowledge, no consensus exists about a system for sorting diagnoses according to subspeciality, and a classification thus had to be made (Table 1; see the complete list and detailed description in supplementary material 1). Part of this was validated by relevant experienced clinicians not part of this study, who stated the most common diagnoses within their field where treatment from a subspecialist gave a significantly better prognosis than treatment from another internist. They also stated diagnoses which they regarded all internists should be able to treat equally well. Afterwards, the chief of medicine at the hospital reviewed the system to ensure it fit the local ways of working.

**Table 1:** The ten most frequent ICD-10 codes in each category of admissions 2005–2008 and 2012–2017

ICD-10 code	Name	Speciality / Subspeciality	Number of Admissions
Category 1 – General internal medicine			
R07	Pain in throat and chest	General internal medicine	986
J18	Pneumonia, unspecified organism	General internal medicine	550
J15	Bacterial pneumonia	General internal medicine	527
I63	Cerebral infarction	General internal medicine	335
N39	Other disorders of urinary system (mainly UTI)	General internal medicine	331
R55	Syncope and collapse	General internal medicine	328
G45	Transient cerebral ischemic attacks and related symptoms	General internal medicine	301
A46	Erysipelas	General internal medicine	184
J20	Acute bronchitis	General internal medicine	162
A41	Other sepsis	General internal medicine	114
Category 2 – Specific for subspecialists in internal medicine			
I48	Atrial fibrillation and flutter	Cardiology	718
J44	Other COPD	Lung Medicine	704
I21	Acute myocardial infarction	Cardiology	566
I50	Heart failure	Cardiology	292
I20	Angina pectoris	Cardiology	263
Z95	Presence of cardiac and vascular implants and grafts	Cardiology	134
R06	Abnormalities of breathing	Lung Medicine	108
R10	Abdominal and pelvic pain	Gastroenterology	102
I47	Paroxysmal tachycardia	Cardiology	101
E11	Type 2 Diabetes Mellitus	Endocrinology	91
Category 3 – Outside internal medicine			
R42	Dizziness and giddiness	Outside internal medicine – ENT	214
F10	Alcohol-related disorders	Outside internal medicine – psych	199
H81	Disorders of vestibular function	Outside internal medicine – ENT	98
R51	Headache	Outside internal medicine – neuro	79
G40	Epilepsy and recurrent seizures	Outside internal medicine – neuro	62
M79	Other and unspecified soft tissue disorders, not elsewhere classified	Outside internal medicine	57
R41	Other symptoms and signs involving cognitive functions and awareness	Outside internal medicine	44
C61	Malignant neoplasm of prostate	Outside internal medicine – urology	36
G43	Migraine	Outside internal medicine – neuro	33
F41	Other anxiety disorders	Outside internal medicine – psych	31

When matching patients with a consultant, it was assumed that most of the treatment was planned and started during the first days of stay, and it was chosen to connect the patients with the consultant(s) on call at the time of admission and/or present during the first 3 days after admission. In situations where there could be two consultants present, the one with the relevant subspeciality was assumed to be in charge of the patient.

To validate whether the classification of consultants was correct, a random sample of admissions with relevant and non-relevant subspecialists was assessed by a hospital physician who was not part of this study. All 25 admissions classified as not having a relevant subspecialist present were correctly classified. For the 25 admissions classified as having a relevant subspecialist present, the name of a relevant subspecialist was not mentioned in four. Only the names of interns or one other subspecialist was mentioned in three of these, and in the remaining one, two non-relevant subspecialists were mentioned. The consequence of misclassification would be less difference between the groups, i.e. a type 2 error (not finding a true effect). Three of the validated admissions coded as not treated by a relevant subspecialist were attended by a relevant subspecialist after day 3 of the admission.

### Variables

There were three outcome variables: readmission, length of stay and in-hospital mortality.

A readmission was identified as a new emergency admission to the same ward within 30 days of discharge from the prior admission for the same patient regardless of diagnosis [17]. Readmissions after certain ICD-codes (I21, I22, I61, I62, I63 and I64) were likely transferred to larger hospitals for specialised treatment (e.g. percutaneous coronary intervention and thrombectomy) and were validated by hospital staff as a transfer back could wrongly be coded as readmission. After the review, 136 of the 206 readmissions were coded as transfers, leaving 70 readmissions.

Length of stay was measured as the time between admission and discharge. In-hospital mortality was identified when the patient was registered as dead upon discharge. Some variables were used to describe the patients and consultants and used as independent variables in multivariable analysis. They included patient and consultant sex, patient age (0–39, 40–59, 60–79 and ≥ 80), nine main diagnostic groups and comorbidities according to the Charlson comorbidity score (0, 1, 2 and ≥ 3 points) [18, 19]. The Charlson comorbidity score was calculated using primary and secondary diagnoses registered at the current and all former admissions during the study period.

### Analysis

All statistical procedures were performed with IBM SPSS Statistics for macOS, version 27 (IBM, Armonk, NY, USA). The patient and consultant characteristics are presented using descriptive statistics. The analysis of the influence of being attended by a relevant subspecialist for the three outcomes was done the same way. First, it was conducted as a descriptive analysis of the bivariable (unadjusted) prospective association between the dependent and independent variable. Then, a multivariable regression analysis was conducted where the models were adjusted for patient age, patient sex, sex of consultant on call at admission, diagnostic group and Charlson comorbidity score. Logistic regression analysis was performed for the dependent categorical variables coded as yes/no, namely readmission and in-hospital death. Odds ratios are reported as adjusted odds ratio (adj. OR) with a 95% confidence interval (95% CI).

Length of stay was analysed using linear regression, and dummy variables were coded for categorical variables with more than two groups (age groups, diagnostic group, and Charlson comorbidity score). Unstandardised coefficients are reported as adjusted coefficients (adj. Coeff.) with a 95% CI.

## Results

**Fig. 1:**
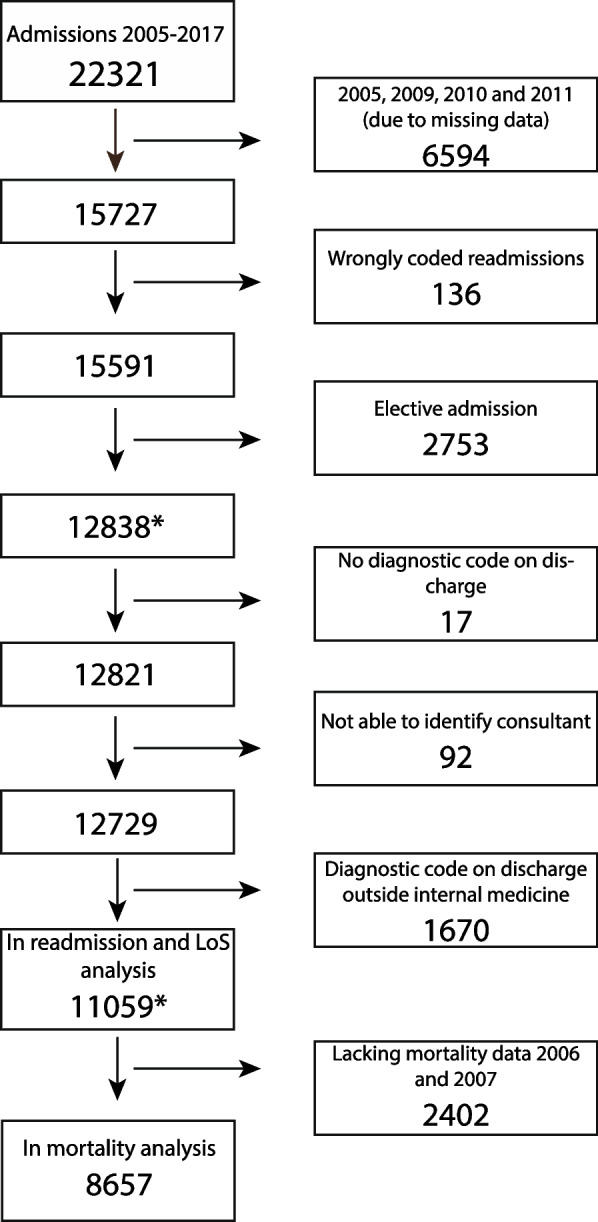
Flowchart of admissions included in the different analysis. * Readmissions were calculated for all emergency admissions (*N* = 12,838), even when the following admission was among the 1799 emergency admissions excluded before including 11,059 admissions

There were 22,321 admissions from 2005 to 2017 (Fig. 1). Of these, 11,059 were included in analyses of readmissions and length of stay, whereas 8657 were included in the analysis of in-hospital mortality. Our data from 2006 to 2007 lacked information about the patients’ state (dead or alive) at discharge, and 2402 admissions could therefore not be analysed for mortality.

### Characteristics of admissions, patients and consultants

Out of the included 11,059 admissions (by 5774 unique patients) with data on readmissions, 5682 (51.4%) admissions were by male patients, and the median age was 70.4 years (SD = 17.2).

In total, 41 consultants were included, out of which 33 were male (Table 2). During the study period, specialists in infectious diseases, oncology, or rheumatology were not present.

**Table 2:** Description of the consultants (*N* = 41)

Characteristics	Consultants	
	Number	%
Sex:		
- Male	33	80.5
- Female	8	19.5
Subspeciality:		
- Cardiology	16	39.0
- Gastroenterology	10	24.4
- Nephrology	2	4.9
- Pulmonology	1	2.4
- Endocrinology	6	14.6
- Haematology	1	2.4
- Other (registrar, generalist, A&E)	5	12.2

### Outcome

Of the 11,059 admissions, 1273 (11.5%) led to emergency readmissions at the same ward within 30 days (Table 3). Those attended by a consultant with a subspeciality relevant to the patient’s diagnosis had an adjusted odds ratio of 0.91 (95% CI: 0.76 to 1.09) of being readmitted.

**Table 3:** Sample characteristics and adjusted odds ratios/coefficients for variables associated with readmissions, in-hospital mortality, and length of stay

Variable	Readmission (*N* = 11,059)			In-Hospital mortality (*N* = 8657)			Length of Stay (*N* = 11,059)		
	N (%)	Adj OR (95%CI)	*P*-value	N (%)	Adj OR (95%CI)	*P*-value	Median	Adj coeff (95%CI)	*P*-value
All	1273 (11.5)			281 (3.2)			2.4		
Attended by relevant subspecialist									
- No (ref)	453 (15.1)	1.00		98 (4.2)	1.00		2.6	1.00	
- Yes	820 (10.2)	0.91 (0.76 to 1.09)	0.308	183 (2.9)	0.71 (0.48 to 1.04)	0.077	2.3	0.18 (−0.07 to 0.42)	0.158
Consultant sex									
- Male (ref)	1009 (11.5)	1.00		211 (3.1)	1.00		2.5	1.00	
- Female	264 (11.6)	1 (0.86 to 1.16)	0.989	70 (3.6)	1.13 (0.85 to 1.5)	0.390	2.2	-0.23* (−0.42 to −0.04)	0.020
Patient sex									
- Male (ref)	657 (11.6)	1.00		136 (3.1)	1.00		2.2	1.00	
- Female	616 (11.5)	1.03 (0.91 to 1.16)	0.633	145 (3.4)	1.17 (0.92 to 1.49)	0.213	2.6	0.11 (−0.02 to 0.29)	0.097
Patient age									
- 0–39 (ref)	69 (9.8)	1.00		1 (0.2)	1.00		1.0	1.00	
- 40–59	191 (11.0)	0.88 (0.65 to 1.18)	0.378	14 (1.0)	3.34 (0.43 to 25.63)	0.247	1.2	0.70* (0.33 to 1.06)	< 0.001
- 60–79	562 (12.1)	0.75* (0.56 to 0.99)	0.041	100 (2.6)	5.88 (0.81 to 42.82)	0.081	2.5	1.86* (1.52 to 2.2)	< 0.001
- ≥ 80	451 (11.5)	0.68* (0.51 to 0.91)	0.009	166 (5.5)	11.80* (1.63 to 85.74)	0.015	3.3	2.61* (2.26 to 2.95)	< 0.001
Diagnostic group									
- General (ref)	529 (9.1)	1.00		132 (2.9)	1.00		2.5	1.00	
- Infectious Diseases	2 (5.7)	0.64 (0.15 to 2.71)	0.543	0 (0.0)	*		2.9	1.2 (−0.21 to 2.6)	0.096
- Cardiology	319 (13.1)	1.43* (1.21 to 1.69)	< 0.001	59 (3.2)	0.87 (0.61 to 1.24)	0.430	1.9	−0.99* (−1.2 to − 0.77)	< 0.001
- Lung Medicine	206 (17.5)	1.67* (1.33 to 2.11)	< 0.001	42 (4.4)	0.91 (0.56 to 1.47)	0.692	3.0	0.22 (−0.11 to 0.55)	0.139
- Gastroenterology	120 (13.5)	1.37* (1.08 to 1.74)	0.009	28 (4.2)	1.09 (0.66 to 1.81)	0.727	2.4	0.36* (0.04 to 0.68)	0.029
- Haematology	49 (29.5)	2.75* (1.86 to 4.07)	< 0.001	12 (9.9)	1.66 (0.79 to 3.45)	0.178	2.8	0.74* (0.05 to 1.43)	0.035
- Endocrinology	9 (4.5)	0.36* (0.18 to 0.71)	0.004	2 (1.4)	0.31 (0.07 to 1.35)	0.119	3.0	1.14* (0.51 to 1.76)	< 0.001
- Nephrology	16 (8.6)	0.8 (0.47 to 1.37)	0.418	1 (0.6)	0.13* (0.02 to 0.94)	0.043	3.9	1.48* (0.85 to 2.11)	< 0.001
- Oncology	14 (28.0)	2.07* (1.07 to 4)	0.030	5 (12.8)	1.66 (0.58 to 4.76)	0.347	5.4	3.10* (1.91 to 4.28)	< 0.001
- Rheumatology	9 (10.7)	1.05 (0.51 to 2.16)	0.903	0 (0.0)	*		3.4	*	
Charlson score									
- 0 (ref)	290 (7.4)	1.00		24 (0.8)	1.00		1.4	1.00	
- 1	335 (9.8)	1.34* (1.13 to 1.6)	0.001	63 (2.4)	2.43* (1.5 to 3.92)	< 0.001	2.6	0.25* (0.06 to 0.44)	0.010
- 2	314 (16.3)	2.33* (1.94 to 2.81)	< 0.001	76 (5.3)	4.96* (3.08 to 7.99)	< 0.001	3.2	0.93* (0.7 to 1.17)	< 0.001
- ≥3	334 (18.3)	2.72* (2.26 to 3.27)	< 0.001	118 (7.8)	7.45* (4.72 to 11.77)	< 0.001	3.9	1.23* (0.93 to 1.53)	< 0.001

*Not included in the model due to few patients with the condition

The Charlson score and some diagnostic groups were most prominently associated with higher readmission in the multivariable logistic regression model. The Charlson score showed a clear gradient also in the adjusted analysis, with a higher score associated with higher readmission rates.

Out of 8657 admitted patients, 281 (3.2%) were discharged as dead. Those attended by a consultant whose subspeciality was relevant to the patients’ diagnosis had an adjusted odds ratio of dying in hospital of 0.71 (95% CI: 0.48 to 1.04).

The Charlson score and age above 80 were significantly associated with higher in-hospital mortality in the multivariable regression model. The Charlson score also showed a clear gradient in the adjusted analysis, with a higher score associated with a higher mortality rate.

The median length of stay for all patients (*N* = 11,059) was 2.4 (interquartile range 1.0–4.9) days. According to the adjusted analysis, patients attended by a consultant with a subspeciality relevant to their diagnosis stayed 0.18 (95% CI: − 0.07 to 0.42) days longer in the hospital. With a clear gradient, increasing age showed the strongest association with length of stay in the multivariable linear regression. Charlson score also showed an association with higher scores leading to more extended hospitalisation. A female consultant on call at admission was associated with shorter stays than when a man was on call (adj. Coeff. − 0.23 (− 0.42 to − 0.04)).

## Discussion

In this study, the direction of the point estimates was towards fewer readmissions and lower in-hospital mortality for those attended by a subspecialist relevant to the patient’s primary diagnosis. Still, this group had stayed somewhat longer in the hospital. Patients with more comorbidities had consistently worse outcomes after the hospital stay regardless of the consultant’s speciality.

### Strengths and limitations

The main strength of this study is that the hospital staffing model made it possible to link the patients and the attending consultants due to the rotation system and few consultants being present at any time.

The main limitation was that no conclusive information about which consultant attended which patient was available, and the study relies on the assumption that the best qualified was the one attending the patient. This is supported by the practice of conferring with relevant subspecialists, which would mean that they have a say without being the attending consultant. Despite the limitations, this method of matching patients to treating physicians is most likely more precise than those used by earlier studies [4, 10, 11, 20].

The diagnosis set at discharge might be affected by the attending consultant. Other studies have adjusted for the physician-diagnosis connection by using an interaction term between physician speciality and principal diagnosis, i.e. whether the interaction between these two variables moderated or modified the outcome [20]. Diagnostic data from Norwegian hospitals have been studied and the accuracy was found to be between 83.5 and 99.8%, depending on the diagnosis [21–24].

Another major limitation could be the matching of subspeciality to diagnosis. To include the full breadth of medical inpatients, a system connecting all diagnostic codes to either an internal medical subspeciality, general internal medicine, or diagnoses outside of internal medicine was made, as no such system was identified. The classification was discussed with experienced clinicians from various specialities and validated with input from academic clinicians not involved in the study. Furthermore, the classification system is transparent as the complete categorisation is available in the supplementary material (Supplementary material 1). However, the model of care or local practice pattern at the hospital in this study might differ from other hospitals, and caution is warranted before applying the findings of this study to other hospitals.

### Does the relevant subspecialist lead to a better outcome?

The direction of the point estimates was towards a lower mortality rate and readmission rate for those attended by a relevant subspecialist but with a somewhat longer stay. This is in line with Weingarten et al. who found a lower mortality odds ratio when treated by a relevant subspecialist [4].

There was no significant effect of treatment from a relevant subspecialist on readmission, mortality, or length of stay, after adjusting for the other variables in the regression model. That is consistent with the findings of McAlister et al. who found no negative association between physicians’ experience and readmission or death and those of Bai et al. who found no significant difference in readmission or mortality when comparing patients treated by generalists and specialists [10, 11]. Bai et al. did, however, find a difference in length of stay that we have not found in this study. The differences found in some disease-specific studies like those from Foody et al. and Jong et al. have not been demonstrated in this study [1, 2].

A measure for disease severity was not available in our study; this might explain why the group attended by a relevant subspecialist had a better outcome in bivariable and unadjusted analyses than in the adjusted analyses.

David Epstein has popularised a theory stating that sub- and subsub-specialisation have gone too far and that the generalist and outsider viewpoints are needed to connect the subspecialists’ deep-but-limited knowledge [25]. His theory applies more to society in general than only to health care. Nevertheless, it might help explain why the subspecialists’ advantage in studies on individual diagnoses seems to be neutralised in the more complex environment in this study. Perhaps the multidisciplinary team consisting of a consultant, a foundation doctor and the nursing staff is, indeed, well-functioning, regardless of the consultant’s subspeciality.

Generalists in the emergency room and subspecialists on the wards might be the trend in larger hospitals with the new specialisation in acute and emergency medicine [26], but that is not an option in smaller hospitals, such as the one studied here. The consultants must cover the emergency room, inpatient wards, and outpatient clinics. It requires them to be broad thinkers in the emergency room, updated on treatment outside their speciality on the ward, and upfront on treatment within their speciality in the clinic. The results in this study with little or no effect of being attended by a relevant subspecialist could be due to the consultants becoming specialised in this way of working.

### Is it the physician, the diagnosis, or other factors?

Age, patient sex and comorbidities are mentioned in the literature as factors affecting the outcome variables of this study [27–30]. Patient sex did not affect the outcomes in this study. Increasing age increased the risk for all outcome variables in unadjusted analyses but did not retain an effect in adjusted analyses. In adjusted analyses for readmission rate, the gradient turned around showing a lower rate with increased age in line with the literature mentioning young age as a risk for readmissions [27]. The most consistent factor associated with the outcomes was the Charlson comorbidities score. It was found to be a clear gradient where a higher level of comorbidity was associated with an increased readmission rate, in-hospital mortality rate and length of stay. Comorbidity is mentioned in the literature as an important factor for all these outcomes [27–30], as it increases complexity and often requires coordination of treatment for the present and the underlying diseases.

The only physician factor analysed was the sex of the consultant. This factor did not affect mortality or readmission rates. However, a shorter length of stay was associated with a female consultant being on call at admission. The most likely reason could be that the female consultants are more stable through the study period, the locum consultants are mainly men, and the female consultants might have been more accustomed to the local practice. However, if this were the case, an effect on readmission and mortality would also be expected.

## Conclusion

In a small-sized hospital where physicians treat patients with a broad spectrum of medical diseases, there is no clear, prospective association between being attended by a relevant subspecialist and a better patient outcome, measured as readmissions, in-hospital mortality, and length of stay. The direction of point estimates was towards lower readmission and in-hospital mortality rates, but these findings are not significant. Taken together, in this hospital at least, there is no argument for changing the staffing policy.

## Supplementary Information

**Additional file 1.**

## Data Availability

The datasets are available from the corresponding author on reasonable request.

## Abbreviations

95%CI: 95% confidence interval
adj. Coeff: Adjusted Coefficient
adj. OR: Adjusted Odds Ratio
coeff: Coefficient
ICD: International classification of diseases
ID: Identification
OR: Odds Ratio
