# Supplementary material for: Relevant vs non-relevant subspecialist for patients hospitalised in internal medicine at a local hospital: which is better? A retrospective cohort study
Source: BMC Health Serv Res. 2022 Nov 14;22:1345. doi: 10.1186/s12913-022-08761-1 (PMC9664716; doi:10.1186/s12913-022-08761-1)
Supplement: Supplementary file 1 — Additional file 1. [file 12913_2022_8761_MOESM1_ESM.docx]

Supplementary material 1 – categorisation of diagnoses:

It was decided to make a list categorising all ICD-10 diagnoses on a three-digit level (excluding decimal codes) according to whether they belong to general internal medicine, the different subspecialties (infectious diseases, cardiology, pulmonary medicine, gastroenterology, haematology, endocrinology, nephrology, oncology and rheumatology) or outside internal medicine. The first author (medical student) made a suggestion. Diagnoses hard to place were discussed with the chief of medicine at the hospital and sorted in line with their practice of allocating patients.

Diagnoses considered to be so common that all specialists in internal medicine should be able to treat them equally good (e.g. D50 – iron deficiency anaemia, J15 – bacterial pneumonia and N39 – other disorders of the urinary system (mainly UTIs)), were coded as general internal medicine [19]. Diagnoses which were difficult to connect to a subspecialty (e.g. E86 - volume depletion and I63 – cerebral infarction) and unspecific diagnoses (e.g. R11 – nausea and vomiting and R07 – pain in throat and chest) were also coded as general internal medicine.

The sorting was validated using input from professors within internal medicine about which diagnoses falling within their subspeciality they felt all specialists in internal medicine should be able to treat equally good and which diagnoses a patient could expect significantly better treatment from a subspecialist.

Patients with cancer/tumours are usually diagnosed and treatment started at other hospitals than the one in this study. When they are admitted to the hospital in this study, the practice is that the organ specialist will be in charge as she/he is the best qualified to treat complications from the cancer and the treatment. Therefore, cancers/tumours were mainly sorted after which organ they have origin in, not as oncological diagnoses.

Diagnoses within each diagnostic category

General Internal Medicine
A04 A05 A08 A09 A35 A36 A37 A38 A39 A40 A41 A46 B00 B01 B02 B06 B07 B08 B09 B25 B26 B27 B30 B37 D50 D51 D52 E40 E41 E42 E43 E44 E45 E46 E50 E51 E52 E53 E54 E55 E56 E58 E59 E60 E61 E63 E64 E65 E66 E67 E68 E86 G45 G47 H34 H60 H65 H66 H67 H92 I26 I61 I62 I63 I64 I65 I66 I69 I80 I81 I82 I83 I84 I85 I86 I87 I88 I89 J00 J01 J02 J05 J06 J09 J10 J11 J12 J13 J14 J15
J16 J17 J18 J20 J21 J22 J30 J93 M53 M54 N30 N39 O25 R02 R03 R07 R45 R46 R50 R52 R53 R55 R56 R57 R58 R59 R60 R61 R62 R63 R64 R65 R68 R69 R74 R77 R78 R79 R80 R81 R82 R84 R89 R93 R94 R96 R98 R99 S06 S20 T4N T51 T68 T78 T81 T88 Z00 Z03 Z04 Z48

Infectious Diseases
A00 A01 A02 A03 A06 A07 A15 A16 A17 A18 A19 A20 A21 A22 A23 A24 A25 A26 A27 A28 A30 A31 A32 A42 A43 A44 A48 A49 A75 A77 A78 A79 A80 A81 A82 A83 A84 A85 A86 A87 A88 A89 A92 A93 A94 A95 A96 A97 A98 A99 B03 B04 B05 B20 B21 B22 B23 B24 B33 B34 B38 B39 B40 B41 B42 B43 B44 B45 B46 B47 B48 B49 B50 B51 B52 B53 B54 B55 B56 B57 B58 B60 B64 B65 B66 B67 B68 B69 B70 B71 B72 B73 B74 B75 B76 B77 B78 B79 B81 B82 B83 B89 B90 B91 B92 B94 B99 G00 G01 G02 G03 G04 G05 G06 G07 G08 R75 R76

Cardiology
C38 D15 I01 I02 I05 I06 I07 I08 I09 I10 I11 I13 I20 I21 I22 I23 I24 I25 I27 I28 I30 I31 I32 I33 I34 I35 I36 I37 I38 I39 I40 I41 I42 I43 I44 I45 I46 I47 I48 I49 I50 I51 I52 I70 I71 I72 I73 I74 I77 I78 I79 I95 I97 I98 I99 R00 R01 Z95

Pulmonary Medicine
C34 D02 D14 D38 D86 E84 J04 J37 J39 J40 J41 J42 J43 J44 J45 J46 J47 J60 J61 J62 J63 J64 J65 J66 J67 J68 J69 J70 J80 J81 J82 J84 J85 J86 J90 J91 J92 J94 J95 J96 J98 J99 R04 R05 R06 R09 R91

Gastroenterology
B15 B16 B17 B18 B19 C15 C16 C17 C18 C19 C20 C21 C22 C23 C24 C25 C26 C48 D01 D12 D13 D19 D20 D37 E73 K20 K21 K22 K23 K25 K26 K27 K28 K29 K30 K31 K50 K51 K52 K55 K58 K59 K62 K63 K64 K66 K67 K70 K71 K72 K73 K74 K75 K76 K77 K82 K83 K85 K86 K87 K90 K91 K92 K93 R10 R11 R12 R13 R14 R15 R16 R17 R18 R19 R85

Haematology
C81 C82 C83 C84 C85 C86 C88 C90 C91 C92 C93 C94 C95 C96 D45 D46 D47 D53 D55 D56 D57 D58 D59 D60 D61 D62 D63 D64 D65 D66 D67 D68 D69 D70 D71 D72 D73 D74 D75 D76 D77 D80 D81 D82 D83 D84 D89 R70 R71 R72

Endocrinology
C37 C73 C74 C75 D34 D35 D44 E00 E01 E02 E03 E04 E05 E06 E07 E10 E11 E12 E13 E14 E15 E16 E20 E21 E22 E23 E24 E25 E26 E27 E28 E29 E30 E31 E32 E34 E35 E70 E71 E72 E74 E75 E76 E77 E78 E79 E80 E83 E88 E89 E90 M80 M81 M82 M83 O24 R73

Nephrology
E87 I12 I15 N00 N01 N02 N03 N04 N05 N06 N07 N08 N10 N11 N12 N14 N15 N16 N17 N18 N19 N25 N26 N27 N28 N29 R31 R33 R34 R35

Rheumatology
E85 I00 M05 M06 M07 M08 M09 M10 M11 M12 M13 M14 M30 M31 M32 M33 M34 M35 M36 M45 M46 M60 M61 M65

Oncology
C39 C45 C49 C76 C77 C78 C79 C80 C97 D21 D36 D48

**Outside internal medicine**

Dermatology
A51 A52 A53 A54 A55 A56 A57 A58 A59 A60 A63 A64 A65 A66 A67 A68 A69 A70 A71 A74 B35 B36 B85 B86 B87 B88 C43 C44 D03 D04 D17 D18 D22 D23 L00 L01 L02 L03 L04 L05 L08 L10 L11 L12 L13 L14 L20 L21 L22 L23 L24 L25 L26 L27 L28 L29 L30 L40 L41 L42 L43 L44 L45 L50 L51 L52 L53 L54 L55 L56 L57 L58 L59 L60 L62 L63 L64 L65 L66 L67 L68 L69 L70 L71 L72 L73 L74 L75 L80 L81 L82 L83 L84 L85 L86 L87 L88 L89 L90 L91 L92 L93 L94 L95 L97 L98 L99 R21 R22 R23

Paediatrics
A33 A50 B80 P00 P01 P02 P03 P04 P05 P07 P08 P10 P11 P12 P13 P14 P15 P20 P21 P22 P23 P24 P25 P26 P27 P28 P29 P35 P36 P37 P38 P39 P50 P51 P52 P53 P54 P55 P56 P57 P58 P59 P60 P61 P70 P71 P72 P74 P75 P76 P77 P78 P80 P81 P83 P90 P91 P92 P93 P94 P95 P96 R95

Gyneacology
A34 C51 C52 C53 C54 C55 C56 C57 C58 D06 D07 D09 D25 D26 D27 D28 D39 N61 N70 N71 N72 N73 N74 N75 N76 N77 N80 N81 N82 N83 N84 N85 N86 N87 N88 N89 N90 N91 N92 N93 N94 N95 N96 N97 N98 N99 O00 O01 O02 O03 O04 O05 O06 O07 O08 O10 O11 O12 O13 O14 O15 O16 O20 O21 O22 O23 O26 O27 O28 O29 O30 O31 O32 O33 O34 O35 O36 O40 O41 O42 O43 O44 O45 O46 O47 O48 O60 O61 O62 O63 O64 O65 O66 O67 O68 O69 O70 O71 O72 O73 O74 O75 O80 O81 O82 O83 O84 O85 O86 O87 O88 O89 O90 O91 O92 O94 O95 O96 O97 O98 O99 R87

Ophthalmology
C69 D31 H00 H01 H02 H03 H04 H05 H06 H10 H11 H13 H15 H16 H17 H18 H19 H20 H21 H22 H25 H26 H27 H28 H30 H31 H32 H33 H35 H36 H40 H42 H43 H44 H45 H46 H47 H48 H49 H50 H51 H52 H53 H54 H55 H57 H58 H59

Orthopaedics
C40 C41 C46 D16 M00 M01 M02 M03 M15 M16 M17 M18 M19 M20 M21 M22 M23 M24 M25 M40 M41 M42 M43 M47 M48 M49 M50 M51 M62 M63 M66 M67 M68 M70 M71 M72 M73 M75 M76 M77 M79 M84 M85 M86 M87 M88 M89 M90 M91 M92 M93 M94 M95 M96 M99 T84

ENT
C00 C01 C02 C03 C04 C05 C06 C07 C08 C09 C10 C11 C12 C13 C14 C30 C31 C32 C33 D00 D10 D11 H61 H62 H68 H69 H70 H71 H72 H73 H74 H75 H80 H81 H82 H83 H90 H91 H93 H94 H95 J03 J31 J32 J33 J34 J35 J36 J38 K00 K01 K02 K03 K04 K05 K06 K07 K08 K09 K10 K11 K12 K13 K14 R42 R43 R49

Breast- and endocrine surgery
C50 D05 D24 N60 N62 N63 N64 R92

Urology
C60 C61 C62 C63 C64 C65 C66 C67 C68 D29 D30 D40 D41 N13 N20 N21 N22 N23 N31 N32 N33 N34 N35 N36 N37 N40 N41 N42 N43 N44 N45 N46 N47 N48 N49 N50 N51 R30 R32 R36 R39 R86

Neurosurgery
C70 C71 C72 D32 D33 D42 D43 G91 I60 I67

Neurology
C47 G09 G10 G11 G12 G13 G14 G20 G21 G22 G23 G24 G25 G26 G30 G31 G32 G35 G36 G37 G40 G41 G43 G44 G46 G50 G51 G52 G53 G54 G55 G56 G57 G58 G59 G60 G61 G62 G63 G64 G70 G71 G72 G73 G80 G81 G82 G83 G90 G92 G93 G94 G95 G96 G97 G98 G99 I68 R20 R25 R26 R27 R29 R40 R41 R44 R47 R51 R54 R83 R90

Gastrointestinal Surgery
K35 K36 K37 K38 K40 K41 K42 K43 K44 K45 K46 K56 K57 K60 K61 K65 K80 K81

Psychiatry
F00 F01 F02 F03 F04 F05 F06 F07 F08 F09 F10 F11 F12 F13 F14 F15 F16 F17 F18 F19 F20 F21 F22 F23 F24 F25 F26 F27 F28 F29 F30 F31 F32 F33 F34 F35 F36 F37 F38 F39 F40 F41 F42 F43 F44 F45 F46 F47 F48 F49 F50 F51 F52 F53 F54 F55 F56 F57 F58 F59 F60 F61 F62 F63 F64 F65 F66 F67 F68 F69 F70 F71 F72 F73 F74 F75 F76 F77 F78 F79 F80 F81 F82 F83 F84 F85 F86 F87 F88 F89 F90 F91 F92 F93 F94 F95 F96 F97 F98 F99 R48
